# The Development of a Framework to Classify Medication Deprescribing Among Patients with Type 2 Diabetes in Primary Care Practices

**DOI:** 10.3390/jcm15072524

**Published:** 2026-03-26

**Authors:** Puja B. Gandhi, Yoav Jacob, Joeita F. MacField, Gia Merlo, Stefanie M. Meyer, Shivani S. Patel, Caroline Rhéaume, Kara L. Staffier, Madeline Watson, Micaela C. Karlsen

**Affiliations:** 1 McGovern Medical School, UTHealth, Houston, TX 77030, USA; puja.gandhi@uth.tmc.edu; 2 Albert Einstein College of Medicine, Bronx, NY 10461, USA; yoav.jacob@einsteinmed.edu; 3 College of Human Medicine, Michigan State University, Grand Rapids, MI 49503, USA; macfiel1@msu.edu; 4 Department of Psychiatry, NYU Grossman School of Medicine, New York, NY 10016, USA; giamerlomd@gmail.com; 5 Department of Nutrition, Dietetics, and Exercise Science, College of Health Professions, Concordia College, Moorhead, MN 56562, USA; smmeyer@cord.edu; 6 Rowan Virtua School of Osteopathic Medicine, Stratford, NJ 08084, USA; 7 Department of Family Medicine and Emergency Medicine, Faculty of Medicine, Université Laval, Quebec City, QC G1V 0A6, Canada; caroline.rheaume@fmed.ulaval.ca; 8 Department of Research, American College of Lifestyle Medicine, Chesterfield, MO 63017, USA; 9 College of Osteopathic Medicine of the Pacific, Western University of Health Sciences, Pomona, CA 91766, USA; 10 Departments of Applied Nutrition and Global Public Health, University of New England, Biddeford, ME 04005, USA

**Keywords:** lifestyle medicine, deprescribing, medication de-escalation, type 2 diabetes, primary care

## Abstract

**Background**: There is growing recognition that certain medical conditions, such as type 2 diabetes (T2D), can be effectively addressed through comprehensive lifestyle changes, thereby reducing reliance on medications; however, little guidance exists on deprescribing following lifestyle change. This study aimed to develop a framework that can be used to better define and standardize across research studies which medication changes in T2D care can be classified as deprescribing. **Methods**: An iterative development process began with a review of medication data exported from electronic health records (EHR) for *n* = 650 patients with T2D, 18–89 years, from two primary care practices with LM board-certified physicians. Included patients were seen during the period of 15 May 2014 to 13 March 2023. All reported T2D medications were grouped into the following categories: insulin, non-insulin, or metformin. A consensus-based review process was employed, facilitated by weekly meetings with the research team, whereby patients were classified as “potentially deprescribed,” “not deprescribed,” or “unclear” (not enough information based on limited, exported EHR data). Patients identified as potentially deprescribed or “unclear” were then further assessed through a more detailed review of their EHR. **Results**: Using the results of this chart review, a framework was developed to identify types of deprescribing, as follows: (1) insulin dose reduced; (2) change from insulin to other non-insulin medication; (3) insulin discontinued; (4) non-insulin T2D medication stopped; (5) dose reduced of the same non-insulin T2D medication; (6) change from any non-insulin medication to metformin or multiple medications + metformin to metformin only; (7) metformin stopped; (8) metformin dose reduced. A total of *n* = 193 patients were identified as having been potentially deprescribed based on the exported EHR data, and after a more detailed review of individual EHR records, 41 were confirmed as deprescribed. **Conclusions**: This study is the first to present a novel framework for classifying deprescribing in the context of positive health outcomes. The framework will facilitate future research evaluating the impact of lifestyle changes on diabetes management and promote comparability across settings for medication outcomes. Future research is needed to apply this framework to quantify deprescribing across various settings with greater precision.

## 1. Introduction

Pharmacological management of type 2 diabetes (T2D) is complex, and medications encompass diverse classes with the most common being insulin and the more common oral and injectables such as sulfonylureas, biguanides, thiazolidinediones, dipeptidyl peptidase-4 (DPP-4) inhibitors, glucagon-like peptide-1 (GLP-1) receptor agonists, and sodium-glucose cotransporter-2 (SGLT-2) inhibitors [1]. According to GoodRx, a cost savings website for prescriptions, metformin (a biguanide) was the number 8 most prescribed medication in the U.S in 2021 [2]. While these medications are often effective at lowering blood glucose levels, they are not without risk, which can range from minor inconveniences to life-threatening complications [3]. For example, sulfonylureas can cause severe hypoglycemia if used in conjunction with other medications or if there is no titration upon improvement in glucose levels, and SGLT-2 inhibitors can lead to a life-threatening euglycemic ketoacidosis [4,5]. Polypharmacy further increases the risk of medication interactions and side effects. Additionally, more complex regimens can lead to worse HbA1c outcomes due to factors such as the complexity of treatment and side effects such as hypoglycemia, and nonadherence to these medications is high, close to 50%, which can lead to poor glycemic control and raise the overall risk for mortality [6,7].

Medication deprescribing, defined as the planned and supervised process of dose reduction or discontinuation of medications that may no longer be beneficial or may be causing harm [8], can alleviate polypharmacy burdens, as well as help to lower healthcare costs [8,9,10]. While the notion of deprescribing medication is not new and is known to be an important aspect of medical care [9,11,12,13,14,15,16,17], little research exists on deprescribing in T2D care necessitated by improvements in health. There is growing recognition that deprescribing of T2D medications following health improvements is possible. Lifestyle medicine (LM) utilizes an evidence-based approach to treat chronic disease [18] and offers enormous potential to achieve such health improvements.

Deprescribing in an LM context is unique in that adequately dosed lifestyle interventions in the form of intensive, therapeutic lifestyle change (ITLC) can produce meaningful and sometimes rapid clinical improvements that reduce the need for pharmacotherapy [19,20,21,22,23]. One area of particular interest is deprescribing among T2D patients, as lifestyle changes can promote not only medication reduction but remission of type 2 diabetes (T2D) [24,25]. Most previous work on deprescribing has been unrelated to health improvements, and a recent qualitative case series by Bradley et al. addressed deprescribing practices among LM physicians, but no standardized, operational framework currently exists to identify which medication changes in T2D management should be considered deprescribing following lifestyle interventions [23].

While clinical guidance and algorithms exist on how to deprescribe, and McGrath et al. summarize tools to identify polypharmacy and assist with deprescribing [9,26], this guidance does not define deprescribing from a research outcomes perspective. The growing interest in LM and subsequent opportunities for deprescribing following lifestyle change highlighted the need for a framework to classify deprescribing in the context of research on health improvements. Furthermore, this topic is timely as clinical guidance increasingly highlights the role of lifestyle change in T2D management. Most significantly, a 2025 clinical practice guideline from the American College of Lifestyle Medicine is the first clinical guideline to feature lifestyle at the center of T2D care, offering 14 practical, evidence-based key action statements (KAS) recommending how to treat T2D with lifestyle, including a KAS on considerations for deprescribing [27]. The United States Preventive Services Task Force and American Diabetes Association recommend lifestyle intervention as the primary treatment modality to slow and reverse the progression of T2D [28].

Deprescribing, which may mitigate potential side effects and improve safety and quality of life for individuals with T2D, is a natural byproduct of lifestyle change in T2D management [29]. Thus, the objective of this study was to develop and pilot test a framework to categorize medication changes as deprescribing vs. non-deprescribing in patients with T2D in primary care, in which the deprescribing was necessitated by improvements in health. This framework is a reproducible classification system that can facilitate comparability of research and guide future evaluations of lifestyle interventions and other research on health improvements. Such future research is needed to fill a gap in the evidence base.

## 2. Methods

This study is based on a retrospective review of electronic health record (EHR) data from two primary care practices that integrate LM with usual care. Individuals seeking care at these practices are not necessarily seeking an LM physician. Both practices employ physicians board certified in LM who offer LM treatment alongside usual care.

Data Source and Extraction: A programmer affiliated with the practices generated a secure, web-based, downloadable dashboard from the EHR, Elation. This dashboard was restricted to *n* = 650 patients aged 18–89 years old with a diagnosis of T2D based on ICD-10 codes who were seen by the practice at least twice during the analysis period of 15 May 2014, through 13 March 2023. The dashboard was securely downloaded as a spreadsheet and contained information on age, sex, documented dates of clinic visits, medication names, strengths, doses, and related physician notes. All data were de-identified prior to analysis.

**Figure 1:**
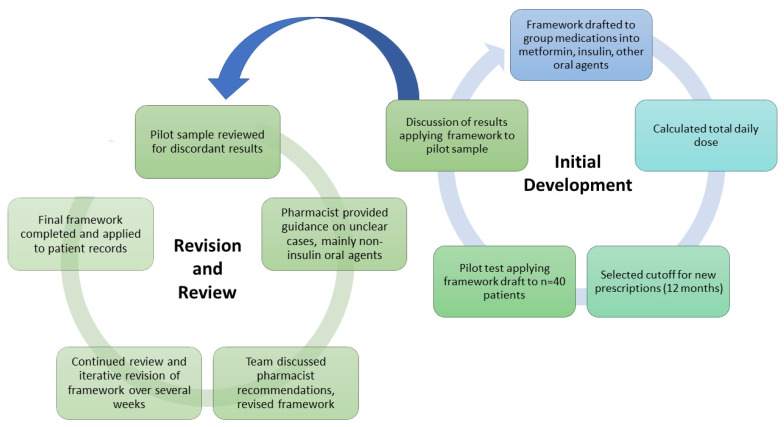
Development process of the deprescribing framework. This figure depicts the iterative process of development and revision.

Initial Data Organization: The study team reviewed the dashboard to determine how to organize the data for relevance on the criteria needed to deprescribe. While the original goal of the study was to identify cases of deprescribing in the records, it became clear that defining deprescribing was difficult in the absence of clear guidelines or a formal framework for its definition. Thus, the team sought to develop a framework by identifying patterns of T2D medication management. The development of the deprescribing framework is summarized in Figure 1.

First, a list of all diabetes medications prescribed in the EHR export was generated, and medications were grouped into the following three broad categories: insulin, metformin, and other (non-insulin/non-metformin) oral diabetes medications. Next, a comprehensive list of specific diabetes medications (brand and generic names) and their common abbreviations was created within each of the three broad categories. A standard approach for calculating daily dose (medication dosage x frequency per day) was agreed upon to facilitate greater ease in understanding patterns of medication management over time. Since both practices require patients to be seen at least annually for refills to be prescribed, as confirmed by the two lead physicians at the practices, we considered patients who had clinical encounters but no medications recorded in the final 12 months of the data period to be possible deprescribing and to be flagged for further review.

Framework Development Process: Because no existing framework for defining T2D deprescribing following improvements in health was available, the team used an iterative, consensus-based process to derive one. The research team consisted of 10 interdisciplinary healthcare professionals, two clinicians with experience in prescribing and deprescribing glucose-lowering medications (PG, CR) and a physician practicing psychiatry (GM), all with additional expertise in LM; two PhDs with expertise in nutrition and LM (MK, SM); 4 medical students (MW, YJ, SP, JM); and one team member with a master’s degree in public health who served as project and data manager (KS).

Pilot Case Review: With this foundation, the entire team worked through approximately 20 patient cases together. During this time, the entire team reviewed the same 20 cases and participated in iterative discussions to ensure alignment in the interpretation of health records and consistency in the classification strategy. Following this, an initial classification framework was developed, and a pilot case review was conducted in which the framework was applied to a sample of 40 patients. Each team member reviewed 10 patients; thus, 2–3 team members reviewed each patient. The team members were blinded to the other reviewer’s responses for each case to reduce bias.

Consensus building: The pilot sample was then reviewed by one team member (KS) to identify any discordant classifications. Although it was clear that moving from insulin to other oral medications constituted deprescribing, there was considerable discordance in classifying changes among various non-insulin oral diabetes medications. A pharmacist with experience in T2D medications was consulted to review the entire medication list and further classify diabetes medications by intensity. The team discussed these recommendations by the pharmacist, and with agreement, adopted them into the categorization scheme.

Iterative Refinement: The team continued to meet weekly to discuss the scheme and develop a deprescribing process, which was revised 2–3 additional times and incorporated ongoing revisions to additional patient records until each member agreed to the protocol. Through these iterative discussions and with the general consensus of our diverse group of medical professionals, the final framework was produced.

Full Application of Framework: All remaining patient records were independently reviewed by at least two members of the 10-member team. Discordant responses were identified and discussed in weekly team meetings until a consensus was reached. All cases identified as deprescribed or possibly deprescribed were advanced for a full EHR review to confirm. This EHR review was conducted by a clinical staff member at one of the practices who had experience working with patients and using the EHR. This staff member closely reviewed information pertinent to deprescribing and medication history, as well as provider notes from relevant encounters, to confirm whether deprescribing occurred.

Ethical Considerations: This study was reviewed by the University of New England Institutional Review Board (IRB #0922-17).

## 3. Results

To develop this framework, the study team reviewed more than 5000 patient encounters from two LM practices to develop a deprescribing framework for T2D medications. Both practices offer LM treatment to all patients, but do not advertise themselves explicitly as “LM practices.” Thus, the deprescribing framework was developed around three broad categories of medications found in general T2D prescribing: insulin, non-insulin/non-metformin oral agents, and metformin. Once general categories were created, the initial data export from the EHR was reviewed, and each patient’s medication changes were classified as deprescribed, not deprescribed, or unclear.

All health care providers on the team agreed that metformin is typically the first medication initiated for patients requiring medical management of T2D and the last medication discontinued during deprescribing in T2D. This is in agreement with the American Diabetes Association’s Standards of Care Guidelines for Diabetes in 2024, which recommends prescribing metformin as first-line therapy and using stepwise addition of other medications with metformin [30]. Within the insulin category, if the insulin dose was reduced (total units of insulin decreased) or if insulin was removed or changed to a non-insulin T2D medication, this was considered deprescribing. Within the non-insulin, non-metformin category, deprescribing was defined as a medication dose reduction, a switch from a non-insulin agent to metformin, or discontinuation of a non-insulin, non-metformin medication. Finally, if the metformin dosage was reduced or stopped completely, this was considered deprescribing.

The ‘not deprescribing’ category was used for the following: lateral change in medications among different non-insulin, non-metformin oral medications; a medication dose increase; or no change in medications. The lateral change category was designated for cases that consisted of multiple transitions between non-insulin oral medications and was not considered deprescribing. This was due to the fact that consistently classifying transitions between different non-insulin, non-metformin T2D medications in a standardized way was not possible, as clinical notes often did not indicate why the transitions were made. A medication increase was identified when there was an increase in the amount of total insulin units or an increase in dosage of non-insulin oral/injectable agents, or when new prescriptions were made that increased in intensity due to medication type—from non-metformin oral agents to insulin, or from metformin to either category.

An ‘Unclear’ category was created to identify more complex cases involving multiple transitions among oral medications. This category was created to flag cases where the categorization was difficult to assess based on the data in the initial, limited EHR data export. Patients initially marked as unclear were sorted into one of the other categories after additional review of the EHR.

**Figure 2:**
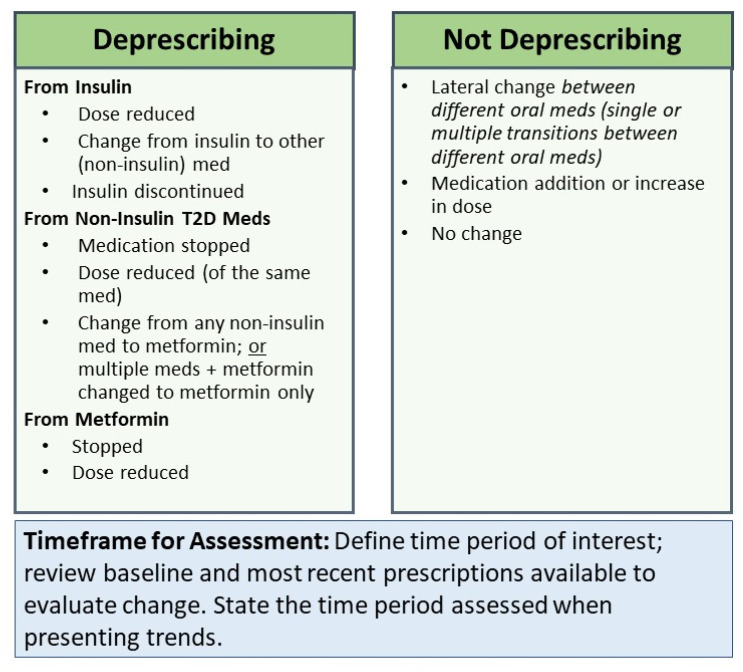
Framework for Identifying Deprescribing of Type 2 Diabetes Medications.

The final framework for categorization of deprescribing included looking at the overall structure of physician prescriptions for patients and categorizing them within the following classes: (1) insulin dose reduced; (2) change from insulin to other non-insulin medication; (3) insulin discontinued; (4) medication stopped; (5) dose reduced of the same medication; (6) change from any non-insulin medication to metformin or multiple medications + metformin to metformin only; (7) metformin stopped; (8) metformin dose reduced. See Table 1 and Figure 2.

If the patient’s last medication was prescribed over one year prior to the end of the data extraction period (13 March 2023), and the patient had a clinical encounter indicating that they were still an active patient, the medications were considered discontinued, and thus it was considered a ‘deprescription’ of medications.

## 4. Discussion

This study developed and piloted a framework to categorize deprescribing of T2D medication for use in a setting where deprescribing was needed due to improvements in health. Despite the existence of clinical guidance and algorithms for clinicians on how to deprescribe [31], little information exists on how to categorize deprescribing in a way that enables detailed and consistent research on deprescribing as an outcome following such health improvements. By explicitly defining categories of deprescribing, this framework provides an operational tool that can be applied to retrospective EHR data and future prospective studies. While this framework can be applied to research in a variety of fields and across a variety of settings, one growing and important area of research that would benefit from such a framework is research on deprescribing when LM is incorporated into medical management.

Our work aligns with and extends prior efforts, including a qualitative study that aimed to describe deprescribing practices among LM practitioners [23]. Unlike their case series, our approach operationalizes deprescribing decisions into explicit categories that can be consistently applied to patient records. This framework may therefore complement existing research on deprescribing in various populations including those in hospice/end-of-life settings [13,14,15], among aging populations and individuals with frailty [12,32], individuals with poorly controlled T2D [33], and individuals post-bariatric surgery [34]. One particular study investigated creating medication categories that could be used as deprescription in the elderly to help prevent symptoms of hypoglycemia [35]. Yet, none of this work has examined deprescribing necessitated in the unique context of health improvements following lifestyle change. This framework provides a foundation for further standardization in research, which will facilitate comparison between studies in the emerging field of LM. Additionally, the research that may be generated using this protocol scheme will allow us to continually identify patterns between intensive LM prescriptions for patients and their increasing pivotal role in deprescribing, thus supporting quality improvement in patient care.

Due to the high level of complexity involved in classifying transitions between different non-insulin, non-metformin T2D medications in a standardized way, we determined that these would be classified as not deprescribing. It is important to acknowledge that clinically, these changes could be beneficial, for example, in terms of reducing pill burden, cost, and/or side effects. The decision to classify these changes as lateral was rooted in the fact that the aim of this framework is to promote standardized research on deprescribing following improvements in health outcomes. The author group overwhelmingly agreed that changes possibly due to side effects or coverage do not fall within this scope; additionally, the framework is intended to facilitate pragmatic understanding of the trajectory of a patient’s medication usage. Part of the utility of the framework is its relative simplicity; the nuances associated with changes from one oral non-metformin medication to another do not consistently contribute to understanding of the bigger picture.

The framework offers three main strengths. First, it was developed through an iterative, consensus-based process involving a multidisciplinary team, supplemented by pharmacist review for pharmacological accuracy. Second, it was pilot-tested and refined using actual EHR data, ensuring applicability in real-world primary care settings. Third, the framework accounts for both dose reductions and discontinuations, as well as transitions between classes, while distinguishing lateral switches and medication escalation. We believe this framework can serve as a template that can promote future research on deprescribing as a result of improvements in health.

Limitations of the study include the relatively small size of the group for consensus (*n* = 10) and the fact that all decisions were made based on the unique experiences of each healthcare professional. Combinations of medications and complex treatment trajectories remain challenging to classify, and some cases require a review of the full EHR for resolution. Although the team was multidisciplinary, not all members had full prescribing expertise. This was mitigated by pharmacist consultation and clinician oversight. The framework was developed based on data from two primary care practices, which may limit generalizability to other care settings. Additionally, while the entire group (*n* = 10) had weekly meetings and extensive discussions to encourage consistency in classifications, and all records were reviewed by at least two team members, no formal inter-rater reliability testing was conducted across the entire group. Finally, though there are evidence-based principles that are applied to medication management in T2D regimens, it should be acknowledged that prescription decisions can also be provider-dependent, as individual practitioners can have a preference for certain medications over others that can make prescribing decisions inconsistent in a real-world setting.

The framework development relied on consensus discussions rather than a formal Delphi or nominal group method, and therefore reflects pragmatic rather than formalized consensus procedures. Despite these limitations, this framework represents a novel contribution by operationalizing the potential for research on deprescribing necessitated by improvements in health. It provides a reproducible method to identify deprescribing events and lays the groundwork for larger studies that can evaluate frequency, predictors, and outcomes of deprescribing in T2D patients who undergo intensive lifestyle change.

## 5. Conclusions

We developed and piloted a framework to classify deprescribing of T2D medications in a primary care setting. This framework is an important step in the ongoing effort to encourage research on LM as a primary modality for treatment of T2D and contributes to standardization in an emerging field and supports the broader integration of LM into diabetes care. Next steps include further validating the framework across diverse populations and settings, and assessing its utility in linking lifestyle interventions to medication deprescribing. Future research may utilize the framework to further assess the efficacy, safety, and clinical significance of lifestyle interventions for the treatment of T2D.

## Figures and Tables

**Table 1:** Definitions of Deprescribing Framework Categories.

Deprescribing	
	From Insulin ο Dose reduced: decrease in total amount of units of insulin over time. ο Change from insulin to another medication: Insulin was stopped, and either a non-insulin, non-metformin T2D medication or metformin was started. ο Insulin discontinued: All insulin was discontinued over time.
	From Non-insulin T2D Meds ο Medication stopped: A non-insulin, non-metformin T2D medication was stopped during the course of prescriptions. ο Dose reduced (of the same medicine): The same non-insulin, non-metformin T2D medication was decreased in dosage over the patient’s prescription course. ο Change from non-insulin T2D medicine to metformin: A non-insulin, non-metformin T2D medication was stopped and switched to metformin of any dose. ο Change from multiple meds + metformin changed to only metformin
	From Metformin ο Stopped: Metformin of any dosage was stopped during their course and not restarted at any point. ο Dose reduced: Total metformin dosing was decreased from beginning of course to most recent data.
Not Deprescribing	
	Lateral change: can be used if there are single or multiple transitions between different non-insulin, non-metformin T2D medications, as we are treating those as equivalent in terms of intensity. Medication increase: there is a clear increase in units of insulin or increase in total dosage of non-insulin medications (including metformin) over time. No change: if they are on the same medication and same dose throughout their course of T2D medication prescription, or if within the first 12 months and last 12 months, the patient ended up on the same dosage, even if there were changes in between.
Unclear	
	Not a final outcome/category—intermediate status; may have been difficult to assess based on limited EHR data export Further review or additional information from the provider needed; best practice is more comprehensive EHR review or to check with the patient’s provider to verify reasons for medication changes or other changes

Specific medications assessed are presented in the Supplementary Materials. After applying the completed framework, 193 patients were identified as potentially deprescribed or unclear. Of these, 41 were confirmed as deprescribed after additional EHR review.

## Data Availability

In accordance with the IRB approval and to ensure confidentiality of patients, data is not available to members outside of the study team.

## Supplementary Materials

The following supporting information can be downloaded at: https://www.mdpi.com/article/10.3390/jcm15072524/s1.

## Abbreviations

The following abbreviations are used in this manuscript:

T2D	Type 2 diabetes
LM	Lifestyle medicine
EHR	Electronic health record
ACLM	American College of Lifestyle Medicine
